# The journey of a lifetime: reflections on my career

**DOI:** 10.3389/fphys.2025.1733333

**Published:** 2025-11-18

**Authors:** Zehava Uni

**Affiliations:** Department of Animal Science, The Robert H. Smith Faculty of Agriculture, Food and Environment, The Hebrew University of Jerusalem, Rehovot, Israel

**Keywords:** poultry (chicken), intestinal development and function, early feeding, in ovo feeding, taste receptor, energy dynamics before hatch, yolk sac tissue

## Introduction

When I was invited to write a reflection on my scientific career, I felt both honoured and deeply moved. This invitation prompted me to look back at more than 35 years of research and teaching, at the Faculty of Agriculture of the Hebrew University, from a broader perspective. It has been a journey that began with my early days as a young PhD student, continued through my postdoctoral work at Cornell University Veterinary School, and evolved into my roles as a Senior Lecturer, Associate Professor, Full Professor, and currently, as Professor Emeritus and Vice President of the World’s Poultry Science Association (WPSA).

These 4 decades of scientific and professional activity have brought me enormous satisfaction, curiosity, and challenges, shaping my identity as a scientist, mentor, colleague, and individual. I have navigated this journey alongside my beloved family, which includes my three children and my life partner.

This reflection is not merely a summary of my academic achievements or contributions to poultry science. It is also a story of meeting poultry students and researchers from various countries, in many conferences, sessions and discussions, sharing mutual enthusiasm to answer research questions and try to solve problems in poultry science. On a side-note, I also hope this story will inspire young female scientists who consider to become an independent researcher and establishing their own laboratories.

Looking back, I recognize that a career in science and research has been the best choice I could have made. Curiosity has continually fuelled my drive to ask new questions, to seek meaningful answers, and what I like best - to transform findings and knowledge into practice. Guiding graduate students and watching them grow into independent researchers has been among the most rewarding aspects of my career.

My research collaborations with poultry scientists worldwide, from United States (mainly North Carolina and Virginia), Brazil, China, Australia, Italy, France, Germany, Poland, and Canada, have broadened my horizons both scientifically and personally. The international knowledge exchanges, whether through joint experiments, laboratory visits, or global conferences, have enriched my perspective and strengthened my belief in the power of global poultry research collaboration. Representing the Hebrew University of Jerusalem to the international research community has always filled me with pride.

## Lessons for young scientists

What advices can I offer to young scientists? My career has taught me few lessons:

- No Guts, No glory

Years ago, I hanged on my office wall, a poster that states, “No guts, No glory”. This statement expressed what I felt during my first years as an independent researcher in the university. I liked the dual meaning of gut/guts (…as I investigated the gut physiology). But obviously, the actual meaning of this statement, is that in order to succeed-you need an inner drive, curiosity, courage, and a touch of adventure.

- Be resilience to difficulties

Reflecting on my personal journey I realize that my determination to become a good researcher is deeply rooted in my family history. Both my parents were the sole survivors of large families which were murdered during the Holocaust. Their survival story was a model for me and implanted in me a sense of duty and responsibility to honour their resilience and strength.

- Follow the direction that sparks your true passion

I extremely enjoyed my biology undergraduate studies as a student at Haifa university. The curriculum combined zoology and botany, regularly conducted in the field, with hands-on learning experiences several times a week. From those days, I learned a life lesson: Follow the path that truly motivates and fulfills you intellectually. You should do what you really love and find meaningful. This principle has guided me ever since, and I share it with all my students.

- Stay flexible to meet research demands and embrace changes in your career journey

My M. Sc. and Ph.D. at the Hebrew University, Faculty of Agriculture, was focused on molecular genetic markers for disease resistance in poultry (under the supervision of Prof. D. Heller and Prof. A. Cahaner). Later, during a postdoctoral fellowship at Cornell Veterinary School (in Prof. Ton Schat laboratory), I expanded my understanding of the avian immune cells and tissue culture. Upon returning to Israel, I was looking for a a tenure-track position in the university. At that time the only available position, in the Animal Science Department, was in the area of poultry nutrition. In those days, the poultry nutrition research was primarily focused on feed ingredients and dietary optimization, with the intestinal system examined mainly through pathology or anatomy. So, I decided to be flexible and integrate my cellular and molecular expertise into poultry nutrition research area.

It was the right decision! Publications that came out from my lab were the first to describe cellular and molecular aspects of intestinal development in poultry. This includes characterizing epithelial cell types: defining chicken enterocytes before and after hatch; studying the proliferation and differentiation patterns of intestinal crypt and villus cells, identifying goblet cells and mucin production; sequencing nutrient transporters in the chicken gut; examined gene expression before and after hatch, and in various early or late nutrition and various environmental conditions; showing the microvilli development and elongation on the apical membrane of the chicken enterocyte cells and also study the effect of early or late feeding (24 h–36 h post hatch) on intestinal cellular dynamics.

- Apply your findings in practice

My Research findings were widely cited and opened new research directions in poultry nutrition. But most importantly, they led to a simple and practical conclusion: there is an opportunity to jumpstart the intestinal maturation and functionality in poultry. Application of this conclusion resulted in “in ovo feeding and early feeding” strategies, currently applied by several industrial companies.

## My research themes

Through my career I had several research themes and interests. Among them are: The pre- and post-hatch morphological, molecularlar and functionalty changes, of the duodenum, jejunum and ileum; The In ovo feeding (feeding the embryo before hatch) and early nutrition interventions; Examine the role of yolk nutrients and yolk sac tissue function during incubation period; Stimulus of feed, specific nutrients (e.g., minerals, vitamin D3) on digestive system development and on bone properties; Identify digestive and absorptive limitations and develop nutritional or management strategies to overcome them; Microflora/microbiome: modulating the gut microbiota in early life; Taste perception in chickens.

More recent work includes energy dynamics in the embryo from mid-incubation until placement, the hatching muscle and breast muscle, use of AI in muscle histology, and early detection of muscle myopathies in poultry. Additional evidences, using new research techniques shows that early feeding promotes intestinal maturation by shifting the ratios of specialized epithelial cells subpopulations.

The following are some key themes from my life-time career which I am honored to present in more details.

### Developmental physiology of the avian digestive system

More than 15 papers were published between 1996 and 2003 summarizing the findings of M. Sc. and PhD students from my lab (Uni et al., 1996; Uni et al. 1998a; Uni et al. 1998b; Uni et al. 1999; Uni et al. 2000; Uni et al., 2023a). Publications characterized localization of enterocyte cells, their turnover at early age, ontogeny of intestinal enzymes, transporters, goblet cells, villi and microvilli structure during the transition from yolk nutrition to exogenous feeding. Also the critical time windows for intestinal maturation and for nutrient absorption was defined as well as the adaptation of gut tissues to nutritional and environmental signals (Geyra et al., 2001a; Geyra et al., 2001b; Gal-Garber et al., 2003; Uni et al., 2000; Uni et al. 2001; Uni et al. 2023b).

The impact of this new body of knowledge provided the conceptual and practical foundation for nutritional management of newly hatched chicks in the poultry industry and led to the idea of early and in ovo feeding (Uni and Ferket, 2004).

### Early nutrition and in ovo feeding

The research identifies key factors limiting the development and survival of pre-hatch broiler embryos and hatchlings, including nutrient availability in the egg, digestive capacity, and reliance on yolk reserves pre and post-hatch. These constraints contribute to poor chick quality and early mortality. Approaches such as early feeding (providing feed immediately post-hatch) and in ovo feeding (nutrient administration before hatch) can mitigate these issues. Combining both methods hold significant potential to improve early growth, feed efficiency, and overall bird performance, especially given to the rapid growth rates of modern broilers.

Introducing and promoting the concept of in ovo feeding, a terminology which refer to nutrient supplementation directly to the embryo amniotic fluid before hatching, was done hand by hand with a great colleague and partner - Prof. Peter Ferket from North Carolina State University. Together we patented the idea (Uni and Ferket, 2003; US Patent No. 6,592,878) and developed few in ovo feeding solutions which are suitable for enriching the amniotic fluid of the embryo 3 days before hatch, during transfer time at the hatchery (Uni et al., 2005; Foye et al., 2007). The pioneering experiments were done with Embrex Inovoject machine who developed the technology for mass injection and for targeting the amniotic fluid of the broiler embryo at E18. Experiments with specific nutrients/minerals and vitamins were preformed successfully. For example, in ovo feeding with zinc-methionine lead to changes in chicken intestinal zinc exporter mRNA expression and to small intestinal functionality (Tako et al., 2005). A formula that contained also carbohydrates improves energy status of late-term chicken embryos and promote hatchability (Uni, et al., 2005; Foye et al., 2007). Exploring in ovo delivery of carbohydrates, amino acids, minerals, and bioactive compounds (see reviews by Kadam et al., 2013; Peebles., 2018; Das et al., 2021) linked early feeding to improved and accelerated intestinal and muscular development (Kornasio et al., 2011), growth, feed efficiency, immune function, and even bone mineralization. For example,: Eggs injected in ovo at E17 with a solution containing minerals, vitamins (including vitamin D3), and carbohydrates produced chicks with improved bone mechanical properties, cortical and trabecular structure, and mineralization at various stages compared to controls. Notably, enhancements were seen pre-hatch and during early post-hatch days, with lasting improvements in bone architecture and mineralization at later stages (d 28 and 54). The results indicate that embryonic nutrition can positively influence both early and long-term skeletal development in broilers (Yair et al., 2013; Yair et al., 2015).

Research from the last 5 years focused on the dynamic changes in the subpopulations of intestinal cells. Using RNAscope methodology we were able to show that nutritional stimulation by in-ovo feeding modulates cellular proliferation, differentiation and maturation in the small intestinal epithelium of pre-post hatch chick by shifting the ratios of specialized epithelial cells (Reicher et al., 2020; Reicher et al., 2022a; Reicher et al., 2022b).

In ovo feeding is now widely used in poultry science. Our publications transformed the understanding of how prenatal nutrition can shape lifetime performance in poultry, influencing both academic research and industrial feeding strategies (Noy and Uni, 2010). An excellent review publication by Oliveira G. et al. (2023) provides a bibliographical mapping of research related to in ovo injection practice and shows the global research from various laboratories using more than 100 different substances and ingredients delivered in ovo. I was glad to see the names of the three most frequently cited authors: Uni (573 citations), Ferket (376 citations) and Peebles (260 citations).

In ovo feeding has taken a great step forward due to the attention of the poultry industry as a practice that can strengthen and leverage poultry production systems.

### The yolk and the yolk sac tissue

The exploration of the chicken embryo nutrition lead to investigation of the yolk - The major source of nutrients for the embryo during the 21 days of incubation. The yolk is composed of nutrient-rich content and surrounded by a tissue (yolk sac tissue = YST) derived from the embryo’s midgut. Our findings pointed towards limitations in yolk mineral (P, Fe, Zn, Cu, and Mn) and fat availability and in their utilization by embryo during incubation (Uni et al., 2012). Content and uptake of minerals in the yolk of broiler embryos during incubation and effect of nutrient enrichment - by in ovo feeding - were studied (Yair and Uni, 2011; Yair et al., 2015) and exhibit positive, long term effects of elevating mineral reserves in the yolk on bone structure, composition, and mechanical properties.

YST functionality was examined by gene expression from E 13 to day of hatch, revealing its dynamic roles in embryonic development (Yadgary et al., 2014). Over 3,500 genes changed expression during this period, reflecting shifts in YST function. Early on, the YST showed high erythropoietic (blood-forming) activity, while later it upregulated genes for lipid digestion, transport, and metabolism. The YST also produced plasma proteins typically made by the liver. Toward hatch, epithelial cell degradation was observed. When we asked the question if incubation temperature has an effect on YST development and functionality, we found that even a variation of less than 2 Celsius (from the optimal incubation temperature) led to impaired functionality. Both “cold” (36.3 °C), and “hot” (39.3 °C) incubation temperatures altered YST gene expression and reduced yolk utilization, impairing yolk utilization and potentially hatchling quality (Dayan et al., 2020). These results demonstrate that non-optimal incubation temperatures disrupt YST metabolic gene regulation, Overall, the YST functions as a multifunctional organ, which function temporarily substituting for the intestine, liver, and bone marrow to support the embryo until hatching (Wang and Uni 2023).

### Taste perception: taste receptors in the chicken intestine

Since curiosity is one of the main characters needed for a researcher, we continued asking questions about the chicken intestine. The question about taste perception in broiler chicken is an important one as it may have an effect on appetite and on feeding behavior. In one of our studies taste receptor genes were identified in the gastrointestinal tract (GIT) of embryonic and post-hatch chickens. Bitter (ggTas2r1, ggTas2r2, ggTas2r7) and umami (ggTas1r1, ggTas1r3) receptors and their signalling proteins were expressed in the gut, suggesting that chickens use taste pathways in the gastrointestinal tract to sense nutrients and regulate digestion. We used a two-choice test to determine taste detection thresholds in chickens and found that chickens were as sensitive to bitterness as mammals but less sensitive to sweet and umami tastes (Cheled-Shoval et al., 2015; Cheled-Shoval et al., 2002).

### Further research activities

Other current research activities in my lab are: efforts for elucidated growth, energy dynamics, metabolic disorders in fast-growing broilers, energy partitioning in the pre-post hatch period and subsequently, in ovo feeding of guanidinoacetate (GAA) supplementation, a precursor for creatine production (an organic compound that facilitates recycling of ATP, primarily in muscle and brain tissue). We also study muscle myopathies (e.g., spaghetti meat, woody breast) and their nutritional determinants, including energy dynamics in developing muscle, and the introducing of AI-assisted histological analysis for early detection of breast muscle tissue abnormalities. (Dayan et al., 2023a; Dayan et al., 2023b; Dayan et al., 2023c).

The impact of this research is via bridging molecular physiology through precision livestock management, offering innovative approaches to meat quality and food loss reduction.

## My career reflection in numbers

- Academic Standing: Full Professor, Department of Animal Sciences, Faculty of Agriculture, Hebrew University of Jerusalem. Personally, I see this as an achievement since the percentage of female full professors in the Israeli academy is relatively low, less than 17% in 2020. This is in comparison to other western countries, like the USA (35%), France (32%) England (31%) and Germany (29%). The fact that representation of women decreases significantly at higher academic ranks - with the percentage of female full professors being lower than the overall percentage of women in senior faculty positions - is a worldwide issue.
- Publications: During my career I published over 100 peer-reviewed publications, 4 book chapters, 14,000+ citations/All these is reflected in h-index 61. https://scholar.google.co.il/citations?hl=iw&user=162oYyUAAAAJ
- Ranking: Ranked among the top Animal Science researchers in Israel and globally. Ranked as number 1 in the list of 100 most cited papers published in poultry science 1945 to 2020 (Taylor Jr, 2021); In 2022 I was placed in the top 0.02% percentage rank for life time achievements in poultry by schalerGPS in the animal science discipline.
- Scientific Influence: Mentor to many MSc and PhD students, most of them are active and involve in academia and industry. Frequent collaborator with universities and industry (among them North Carolina State University, Wageningen University, Virginia Tech, Embrex, Evonik, Zinpro). Speaker and organizer in global poultry and animal nutrition symposia. Vice-President of the World Poultry Science Association (WPSA)

In summary, by integrating cellular and molecular knowledge into applied poultry nutrition, we demonstrated that scientific insight could lead to practical advances for the industry. The new body of knowledge led to a revolution in poultry nutrition. The understanding that intestinal functionality can be affected during a specific window time of “pre-post hatch period” had a pivotal effect on global chicken performance.

I wish to thank my mentors along my career: Avigdor Cahaner, Dan Heller, Sue Lamont, Ton Schat, Paul Siegel, Peter Ferket, Catherine Ricks and Erik Wong. All of them were an inspiration to my academic and research life.

My scientific path has been shaped by curiosity, perseverance, and a passion for connecting ideas, people, and generations. I feel immense gratitude for having contributed to poultry science, to the progress of my students, and to the worldwide community of poultry researchers.
